# Stable tetraploid olive (*Olea europaea* L.) plants derived from embryogenic cultures show altered growth and improved water retention

**DOI:** 10.3389/fpls.2026.1941427

**Published:** 2026-10-13

**Authors:** Laia Ribalta, Fernando Palencia, Isabel Narváez, Azahara C. Martín, José Ángel Mercado, Fernando Pliego-Alfaro, Elena Palomo-Ríos

**Affiliations:** 1 Instituto de Hortofruticultura Subtropical y Mediterránea “La Mayora” (IHSM-UMA-CSIC), Departamento de Botánica y Fisiología Vegetal, Universidad de Málaga, Málaga, Spain; 2 Instituto de Agricultura Sostenible (IAS-CSIC), Departamento de Mejora Genética Vegetal, Córdoba, Spain

**Keywords:** chromosome doubling, colchicine, drought tolerance, flow cytometry, genetic improvement, *in vitro* polyploidization, somatic embryos

## Abstract

The olive tree (*Olea europaea* L.) faces increasing challenges to sustainability and genetic improvement due to climate-change-driven water scarcity and heightened biotic stress from emerging pests and diseases. These challenges are exacerbated by the shift towards high-density and super-intensive production systems. Its prolonged juvenile phase and narrow genetic base further complicates its situation. In this context, artificial polyploidization, particularly autotetraploid induction, represents a powerful biotechnological strategy to enhance plant plasticity, architectural compactness, and stress resilience. A chromosome-doubling protocol was established and optimized using embryogenic callus cultures (cv. ‘Picual’, line P1), from which somatic embryos were exposed to 0.1% colchicine for 1–6 days to induce autotetraploidy. Ploidy levels were assessed by flow cytometry and confirmed by chromosome counting, and stable tetraploid lines were characterized morpho-anatomically and physiologically under *in vitro* conditions and after 6 months and three-four years of greenhouse growth. The optimal treatment (2–3 days) enabled efficient recovery of stable, non-chimeric tetraploids (4n = 92). Tetraploid plants exhibited a pronounced gigas effect, with wider, thicker leaves and increased cell size across tissues, along with reduced stomatal density. Morphologically, tetraploids exhibited a more compact growth habit and slower development than diploids. At the physiological level, they maintained stable photosynthetic performance and displayed improved leaf water retention in desiccation experiments. Our results demonstrate, for the first time, that embryogenic cultures provide an efficient platform for the recovery of stable, non-chimeric tetraploid olive plants. These plants represent a valuable genetic resource for investigating the effects of genome duplication and may facilitate the future development of cultivars and rootstocks with enhanced resilience to environmental stress.

## 1 Introduction

The olive tree (*Olea europaea* L.) is a key crop in the Mediterranean basin, with major economic, nutritional and cultural importance (Diez et al., 2015). However, olive cultivation is increasingly challenged by climate change-driven water scarcity, emerging pests and diseases, and the expansion of high-density production systems (Guerrero-Casado et al., 2021; Sobreiro et al., 2023). These challenges, have increased the need for genetic improvement aimed at developing cultivars and rootstocks better adapted to a changing environment and the new intensive production conditions. Conventional olive breeding is particularly slow, as the prolonged juvenile phase limits the rapid evaluation of progenies: e.g., approximately two decades may be required to release new cultivars (León et al., 2023). Moreover, the use of wild olive germplasm as a source of stress resistance can introduce undesirable agronomic traits and requires extensive backcrossing to recover the desired phenotype (Klepo et al., 2014). Indeed, only 13 cultivars were released from olive breeding programs between 1992 and 2019 (León et al., 2023), highlighting the need for complementary strategies to accelerate genetic improvement.

Artificial polyploidization is a promising strategy to enhance plants’ plasticity (Sattler et al., 2016; Niazian and Nalousi, 2020). Chromosome doubling can induce changes in cell size, plant architecture, leaf anatomy, physiology, and stress responses, collectively known as the gigas effect (Dhooghe et al., 2011; Trojak-Goluch et al., 2021). In particular, tetraploids may exhibit reduced growth and enhanced responses to abiotic stress, making them potentially valuable as cultivars or rootstocks in intensive horticultural systems (Ruiz et al., 2020; Touchell et al., 2020; Da Silva Costa et al., 2025). *In vitro* chromosome doubling is widely used because it allows polyploid induction under controlled conditions, although its efficiency strongly depends on the explant, antimitotic agent, treatment conditions, and regeneration system. Furthermore, the generation of mixoploid or chimeric tissues is a major limitation, making reliable ploidy verification and the recovery of stable polyploid plants essential (Dhooghe et al., 2011; Sattler et al., 2016).

Polyploid cytotypes have been described in certain wild subspecies of olive; however, these ploidy levels are not present in the germplasm of cultivated olive. In commercial varieties of *Olea europaea* subsp. *europaea*, the stable and predominant chromosomal state is x = 23; therefore, a diploid individual has 2n = 2x = 46 chromosomes, and spontaneous tetraploid forms (4n = 92) do not occur naturally in cultivated olive populations (Rugini et al., 2016). Consequently, the experimental generation of tetraploid genotypes in cultivated olive represents a strategy to introduce novel variability and to evaluate their potential use in breeding programs. Tetraploid olive plants were obtained from *in vitro* shoot-tip fragmentations of a mixoploid plant obtained through gamma irradiation of *in vitro* cuttings of ‘Leccino’ and ‘Frantoio’ cultivars (Pannelli et al., 1990; Rugini et al., 1996). These plants exhibited compact, reduced growth habits with large, thick, ovate leaves and larger floral cells, although fruit size was not affected (Caporali et al., 2014). More recently, oryzalin treatment of *in vitro* uninodal cuttings of ‘Moraiolo’ cultivar induced some morphological modifications, but ploidy level was not assessed, preventing confirmation of stable chromosome doubling (Ozair et al., 2014). Thus, an efficient approach for the reproducible generation and recovery of stable, non-chimeric tetraploid plants in cultivated olive is still lacking.

The present study establishes and optimizes an efficient and reliable embryogenic culture-based protocol for chromosome doubling in cultivated olive using colchicine and evaluates the stability of the resulting tetraploid plants. Their growth pattern, leaf morphology and anatomy, and water-retention capacity have been characterized to determine the phenotypic and physiological consequences of genome duplication. The plants obtained represent a novel genetic resource for investigating polyploidy in olive and for exploring its potential applications in breeding programs and in the development of rootstocks adapted to intensive production systems and environmental stress.

## 2 Materials and methods

### 2.1 Plant material and culture conditions

Olive embryogenic callus, line P1, originated from the radicle of a mature zygotic embryo of cv. ‘Picual’ following the method outlined by Orinos and Mitrakos (1991), was used in this work. The embryogenic line was maintained on ECO medium for cyclic olive embryogenesis (Pérez-Barranco et al., 2009), which contained OM macroelements (Cañas and Benbadis, 1988), ¼ MS microelements (Murashige and Skoog, 1962), ½ OM vitamins, 50 mg L^−1^ myo-inositol, 20 g L^−1^ sucrose, and 550 mg L^−1^ glutamine, supplemented with 0.25 µM indole-3-butyric acid (IBA), 0.44 µM 6-benzyladenine (BA), 0.5 µM 6-dimethylallylamino purine (2iP), 200 mg L^−1^ cefotaxime and 1 g L^−1^ caseine hydrolysate. The medium was solidified with 3 g L^−1^ phytagel (Sigma P8169). Cultures were kept in the dark at 25 ± 2 °C and subcultured onto fresh medium every 4 weeks.

### 2.2 Colchicine treatment

To identify colchicine concentrations and exposure times capable of inducing chromosome doubling in somatic embryos (SE) while maintaining an acceptable level of tissue viability, a range of treatments was selected based on preliminary assays (Palencia, 2019). Embryogenic calli were cultured in ECO liquid medium for 3 weeks in a shaking incubator at 120 rpm. The resulting material was passed through a 2 mm mesh to isolate a highly homogenous fraction consisting predominantly of globular-stage SE, approximately 1.0 to 1.5 mm in diameter, which were used for assays. From this fraction, 0.5 g of globular SEs were transferred to flasks containing 25 mL of ECO liquid medium supplemented with 0.05 or 0.1% (v/v) colchicine solution (Colchicine BioChemica, A4082, PanReac) for 1, 2, 3, and 6 days, aiming to identify a treatment close to the median lethal dose (LD_50_). The colchicine stock was prepared by dissolving the compound in ethanol at 0.5% (w/v) and stored at 4 °C. As a control, globular SEs were cultured on colchicine-free ECO liquid medium. After treatment, isolated globular SEs were transferred to standard solid ECO medium for recovery in darkness. After three successive subcultures of 4 weeks each, the embryogenic material derived from a single surviving SE was considered and maintained as an independent line.

SE survival rate after colchicine treatments and polyploid induction efficiency were evaluated. Polyploid induction efficiency was measured with the method described by Bouvier et al. (1994), and expressed as a percentage using the following formula:


$$Efficiency\;\left(\%\right)=\frac{Number\;of\;poliploids}{Number\;of\;diploids}\times Survival\;rate\;\left(\%\right)$$

Efficiency ranges from 0 to 100; 100 indicates that all treated calli survived and showed polyploidy. Zero would indicate that all treated calli died or no polyploidy induction occurred.

### 2.3 Plant regeneration

Embryogenic material derived from SE that survived the colchicine treatment was used for plant regeneration following the protocol described by Cerezo et al. (2011). SE were matured for 4 weeks on basal ECO medium lacking growth regulators and cefotaxime, and supplemented with 1 g L^−1^ activated charcoal. Then, the embryos that had turned white-opaque were transferred to the same medium with a cellulose acetate dialysis membrane (MWCO 12,000 Da; Sigma D9777) placed on the surface and cultured for an additional 4 weeks. Dialysis membranes were prepared according to the manufacturer’s instructions and autoclaved twice in distilled water at 121 °C for 20 min. For germination, mature embryos at the cotyledon stage, measuring more than 5 mm, were cultured for 12 weeks on a modified Murashige and Skoog (MS) medium containing 1/3 of the macroelements and 10 g L^−1^ sucrose. Subsequently, emerging shoots were multiplied on RP medium (Roussos and Pontikis, 2002), supplemented with 1–2 mg L^−1^ zeatin riboside (Vidoy-Mercado et al., 2012).

For rooting, apical shoots with two nodes were cultured on tubes containing 3 ml of basal RP medium supplemented with 10 mg/L IBA for three days; afterwards, shoots were transferred to RP solid medium without auxin but supplemented with 1 g/L activated charcoal (Narváez et al., 2018). Rooted shoots were acclimatized to ex vitro conditions in jiffy trays with peat moss: perlite (1:1) during 6 weeks, and fertilized with 0,5X Hoagland´s solution weekly. Afterwards, they were transplanted to 1 L pots with 70% Projar Professional substrate (Projar Group), 30% sand and 2 g osmocote/pot. During the growth season, plants were irrigated weekly with a nutrient solution containing: 2 g/L Soluble Nitrophoska 12-5-30, 1 g/L Calcium nitrate, 1 g/L Sequestrene. In addition, twice in the growth season, 2 ml/L of the biostimulant Isabión (Singenta) were also added. After the first year, plants were transplanted to 2 L pots with the same type of substrate and fertilization regime. Due to the olive bushy growth habit, plants were regularly pruned leaving a main axis and some lateral branches. Plants were grown in a confined greenhouse with a cooling system, 30 °C maximum temperature, and daylight conditions.

### 2.4 Ploidy analysis by flow cytometry

To determine the relative nuclear DNA content of embryos and leaves from *in vitro*-regenerated plants obtained from the embryogenic lines recovered after the treatments, flow cytometric analyses were performed using a PA-II Ploidy Analyzer (Partec, Münster, Germany) equipped with a mercury arc lamp for fluorescence excitation, and the CyStain™ UV Precise kit. Approximately 0.5 cm^2^ of tissue (several embryos together) or 0.2 g of leaf material were placed in a glass Petri dish, and 3–4 drops of the Nuclei Extraction buffer (0.5 mL) were added. The tissue was finely chopped with a razor blade for 30–60 s, after which the remaining extraction buffer was added and the suspension was incubated for approximately 1 min. The resulting suspension was filtered through a 30 µm CellTrics™ filter (Ref 04-0042-2326) into a sample tube (Ref 04-2000). Subsequently, 2 mL of Staining Buffer was added, and the samples were analyzed after 30–60 s.

The stained nuclei were detected according to their DAPI fluorescence intensity, and the resulting data were displayed as one-parameter histograms of relative fluorescence intensity. The flow cytometer was operated on a linear scale, and calibration was performed by adjusting the G_0_/G_1_ peak of the diploid reference line (P1) to channel 50 (corresponding to 2C relative DNA content). Consequently, stable tetraploid genotypes were identified by the shifting of their main peak to channel 100 (corresponding to 4C relative DNA content). About 500–1000 nuclei were measured per sample.

### 2.5 Chromosome counting

For somatic chromosome counting, *in vitro*-rooted plants were used; once roots had reached a minimum length of approximately 1 cm, they were excised and used to prepare mitotic metaphase spreads. In some cases, root growth *in vitro* was suboptimal or showed low mitotic activity; in these instances, additional root samples were collected from established genotypes grown in pots under greenhouse conditions.

In all cases, chromosome spreads were prepared as previously described (Kato et al., 2004; Rey et al., 2018), with some modifications. Briefly, root tips were treated with nitrous oxide gas for 4 h to accumulate cells at mitotic metaphase. Subsequently, the treated roots were fixed in freshly prepared 3:1 (v/v) absolute ethanol:glacial acetic acid for 24 h at room temperature and then stored in 70% ethanol at -20 °C until use. After washing in ice-cold water, root apices (containing actively dividing cells) were dissected and enzymatically digested in a solution containing 1% pectolyase Y23 and 4% cellulase Onozuka R-10 (Yakult Pharmaceutical, Tokyo, Japan) in 1X citrate buffer for 1 h 30 min at 37 °C. Following digestion, the material was washed in 70% ethanol, mechanically dissociated with a needle into a fine cell suspension, and centrifuged to remove the ethanol. The resulting pellet was resuspended in 100% acetic acid, and 7 µl of the suspension was dropped onto a glass slide for chromosome spreading.

Chromosomes were counterstained with DAPI (1 μg/ml) and mounted in Prolong Diamond (Thermo Fisher Scientific Molecular Probes, Eugene, OR, USA). Images were acquired using a Leica DM5500B microscope equipped with a Hamamatsu ORCA-FLASH4.0 camera and controlled by Leica LAS X software v2.0.

### 2.6 Morphological and anatomical characterization of tetraploid plants

Three tetraploid lines (31, 33, and 44) were selected for morpho-anatomical evaluation following robust ploidy verification via flow cytometry in both callus and leaf tissues, which confirmed the absolute absence of mixoploidy or chimerism. To assess *in vitro* performance, 20 uniform shoots per autotetraploid genotype, alongside the diploid control (P1), were micropropagated on RP medium over three consecutive subcultures at 8-week intervals. During this period, number and length of axillary shoots were systematically recorded.

Later, six-month-old diploid and tetraploid plants growing in the greenhouse were compared for morphological traits, including plant height, internode length and stem diameter with 15 plants per line. Thirty mature leaves were randomly sampled from diploid and tetraploid plants, and leaf area, leaf length and width were assessed using a flatbed scanner VIISAN VF3240 (Beijing VIISAN Technology Co., Ltd).

A stomatal analysis was also carried out using 10 mature leaves randomly sampled and collected from 9:00 to 11:00 a.m. First, the surface was cleaned with distilled water and 70% ethanol. The abaxial surface was gently cleared of peltate trichomes using adhesive tape. Afterwards, a thin layer of transparent nail polish was applied to obtain epidermal impressions; after drying, the imprint was lifted using transparent adhesive tape, mounted on microscope slides, and examined under a microscope.

Plants were maintained in the greenhouse over consecutive years. During the third and fourth year, additional anatomical and physiological studies were undertaken to further characterize the tetraploid lines. For the anatomical leaf analysis, lamina thickness was measured in 10 fully expanded leaves per genotype using an electronic digital micrometer (Palmer, 0–25 mm). Measurements were taken at the central region of the leaf blade, avoiding the midrib. To investigate the anatomical basis of differences in leaf thickness among genotypes, histological analyses were performed on leaf cross-sections. For this purpose, 5 × 3 mm leaf segments were collected from the central portion of each leaf, avoiding the midrib, from three independent leaves per line. Samples were fixed in PEM buffer (100 mM PIPES, 10 mM EGTA, 10 mM MgSO_4_, pH 6.9) containing 4% (w/v) paraformaldehyde overnight at 4 °C. After fixation, tissues were washed, dehydrated through a graded ethanol series, and infiltrated with acrylic resin (London White) by gradually replacing ethanol with resin of increasing concentration. After polymerization, semi-thin sections (1 µm) were prepared using a UC7/FC7 ultramicrotome and mounted on Vectabond^®^-coated microscope slides to improve tissue adhesion. Sections were subsequently stained with 0.05% (w/v) Toluidine Blue O for 10 min to enhance tissue contrast and facilitate visualization of leaf structure. In these histological sections, tissue thickness was determined for the cuticle, adaxial epidermis, palisade parenchyma, spongy parenchyma, and abaxial epidermis. In addition, the cross-sectional area of adaxial and abaxial epidermal cells, as well as palisade and spongy parenchyma cells, was quantified. For each line, three independent leaf samples were analyzed, and ten measurements were recorded per anatomical parameter and sample. All image processing and quantitative measurements were performed using FIJI software (ImageJ, version 1.53p).

### 2.7 Physiological characterization

Photosynthetic performance was evaluated using Pulse Amplitude Modulation (PAM, Junior-PAM, Walz, Germany) fluorometry to characterize the functional status of photosystem II (PSII) in diploid and tetraploid olive plants. All measurements were conducted on fully expanded, healthy leaves from the upper canopy, ensuring developmental homogeneity and comparable light exposure among samples; one leaf from each of seven independent plants per line was analyzed. Leaves were adapted to darkness for 30 min prior to the analysis and the maximum quantum efficiency of PSII rate (Fv/Fm), the apparent quantum efficiency (α) and the electron transport capacity (ETR) at irradiance of 825 µmol m^-2^ s^-1^ were obtained.

Stomatal conductance (gs) was also measured using a portable leaf porometer (SC-1, Decagon Devices Inc., Pullman, WA, USA). Measurements were performed on three fully expanded leaves per plant from seven plants per genotype between 12:00 and 15:00 h.

### 2.8 Leaf desiccation assay

To assess a leaf desiccation experiment, 14 fully expanded leaves were collected from each of the control and tetraploid lines. Individual leaf area was determined from scanned images using ImageJ, afterwards the initial fresh weight (FW0) was recorded. Leaves were rehydrated for 24 h at 24 °C under indirect light, with their petioles immersed in distilled water, to obtain the turgid weight. Based on their initial fresh weight, leaves were allocated into two groups (n=7) with comparable weight distributions to ensure similar starting conditions between treatments. One group was maintained in sealed containers over a saturated KNO_3_ solution (93% relative humidity; high-RH treatment), while the other was exposed to a saturated MgCl_2_ solution (32% relative humidity; low-RH treatment). In both treatments, leaves were placed on a support to prevent direct contact with the salt solution and incubated at 24 °C under indirect light. Fresh weight was recorded after 48 h of treatment (FW48). Leaves were subsequently oven-dried at 85 °C for 72 h to obtain the dry weight (DW).

Relative water content (RWC) at 48 h was calculated as:


$$RWC\;\left(\%\right)=\frac{\left(FW48-DW\right)}{\left(TW-DW\right)}\times 100\;\left(\%\right)$$

From these measurements, additional traits describing leaf water loss dynamics were derived, including water loss rate (WLR), calculated as the fresh weight loss after 48 h of dehydration per unit leaf area, and leaf mass per area (LMA), calculated as the ratio of leaf dry mass (DW) to leaf area (mg cm^−2^).

### 2.9 Statistical analysis

Statistical analyses were performed using GraphPad Prism version 8.4.3 and RStudio. Data normality and homogeneity of variances were assessed using Bartlett’s and Brown–Forsythe tests. For morphological and anatomical traits where parametric assumptions were met, differences among genotypes were evaluated using one-way ANOVA followed by Tukey’s HDS *post hoc* test. However, for *in vitro* multiplication parameters (number and length of axillary shoots), which exhibited significant heteroscedasticity (Brown-Forsythe, P< 0.0001), a robust Welch’s ANOVA was applied, followed by the Games-Howell *post hoc* test to account for unequal variances and sample size.

The allometric relationship between plant height (H) and stem diameter (D) was statistically analyzed using the smatr package in R. Standardized major axis (SMA) regressions were fitted on base-10 log-transformed data using the linear model:


$$\log_{10}\left(H\right)=\alpha +\beta \log_{10}\left(D\right)$$

where β represents the scaling exponent (slope) and α the elevation parameter (intercept). Scaling models were first fitted separately for each genotype. Homogeneity of slopes across genotypes was evaluated using likelihood ratio tests. Where slope homogeneity held, a common slope model was fitted to test for elevation differences (intercept shifts) among genotypes. Pairwise *post-hoc* comparisons for both slopes and elevations were conducted with P-value adjustments for multiple testing.

For physiological data involving multiple experimental factors and their interactions, a two-way ANOVA followed by Sidak’s multiple comparisons test was employed.

All results are presented as mean ± SD, except height and stem diameter for their allometric relationship study, presented as mean ± SE: for all, the level of statistical significance was set at p< 0.05. To ensure consistency and accurately reflect biological variability, particularly in instances where initial rounding resulted in standard deviation values of “ ± 0.0”, precision was increased to two or three decimal places across the corresponding data tables.

## 3 Results

### 3.1 Effect of colchicine and exposure time on ploidy induction in embryogenic calli

The lower colchicine concentration tested (0.05%) resulted in elevated SE survival rates (circa 80%; data not shown) but failed to induce chromosome doubling, indicating insufficient antimitotic activity at this dose. In contrast, treatments with 0.1% colchicine showed a clear time-dependent effect on both embryo survival and polyploidization efficiency (**Table 1**). Survival rates dropped significantly as exposure time increased, from 83.3% after 1 day to a critical 6.5% after 6 days. Intermediate exposure periods of 2 and 3 days resulted in survival rates of 44.2% and 39.5%, respectively, successfully establishing conditions close to the LD_50_ threshold.

**Table 1:** Survival rate of SE from P1 olive embryogenic line and tetraploid induction rate of recovered lines 3 months after colchicine treatment (0.1% (v/v) at different time periods). Tetraploid nature was assessed by flow cytometry.

Colchicine treatment duration (days)	No. of treated SE	Surviving SE (%)	No. of analyzed lines	Polyploid lines (%)
1	150	83.3	–	–
2	397	44.2	177	2.8
3	294	39.5	118	9.3
6	240	6.5	16	12.5

Flow cytometry screening of the recovered calli, using the non-treated P1 line as a diploid reference, showed distinct fluorescence histograms. Under linear scale calibration, diploid nuclei produced a single sharp peak at channel 50 (2C), whereas stable tetraploid calli exhibited a corresponding peak at channel 100 (4C), enabling unambiguous ploidy discrimination (**Figure 1**). The proportion of polyploidy lines among the survivors increased with exposure duration, ranging from 2.8% at 2 days at 12.5% at 6 days (**Table 1**), indicating that longer exposure enhances chromosome doubling efficiency despite reducing survival rate.

**Figure 1:**
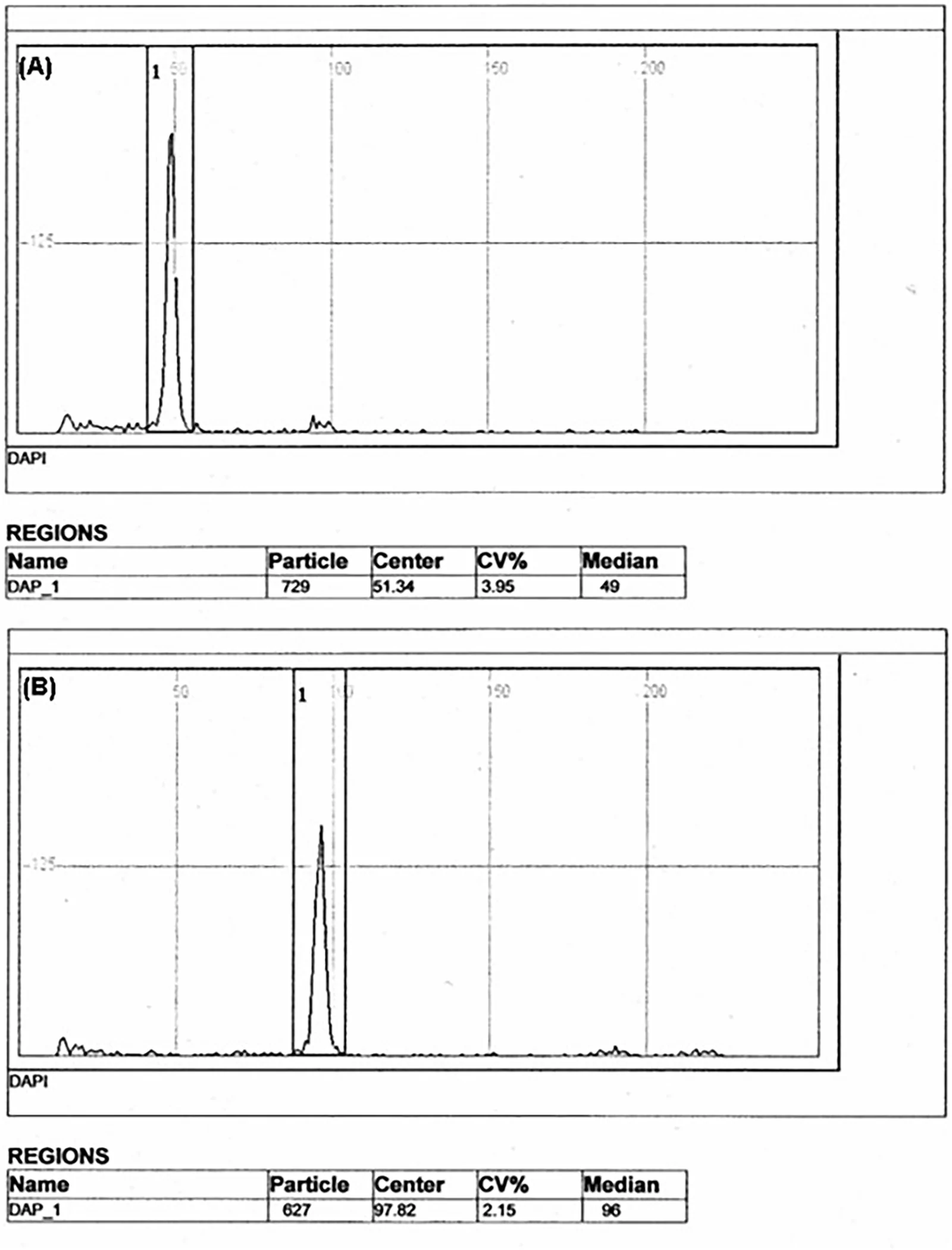
Flow cytometry histograms for two olive embryogenic lines. **(A)** Control diploid line P1, 2n. **(B)** Tetraploid line 31, 4n.

### 3.2 Recovery of polyploid olive plants

The embryogenic callus lines, putatively tetraploid, were maintained under proliferation conditions for several months to evaluate their long-term stability. A second flow cytometry assessment was then performed by subdividing each callus into 4–6 independent sectors (**Figures 2A, B**) to uncover potential heterogeneity. This analysis revealed that some lines maintained the tetraploid trait across all sectors, while others displayed mixed profiles, with regions containing either 4n or 2n nuclei. These findings confirmed the presence of sectorial chimerism in lines initially identified as uniformly tetraploids. Interestingly, in lines 31, 33, and 44 from the 2-day treatment, both the original callus and all subsequently analyzed sectors consistently exhibited a homogeneous tetraploid profile, with no evidence of diploid presence, indicating stable chromosome doubling in these lines. Therefore, these three lines were subjected to SE maturation and germination to regenerate plants (**Figure 2C**). *In vitro* plant stocks were successfully established from the recovered plants (**Figure 2D**), whose ploidy level was confirmed in leaf tissue by flow cytometry.

**Figure 2:**
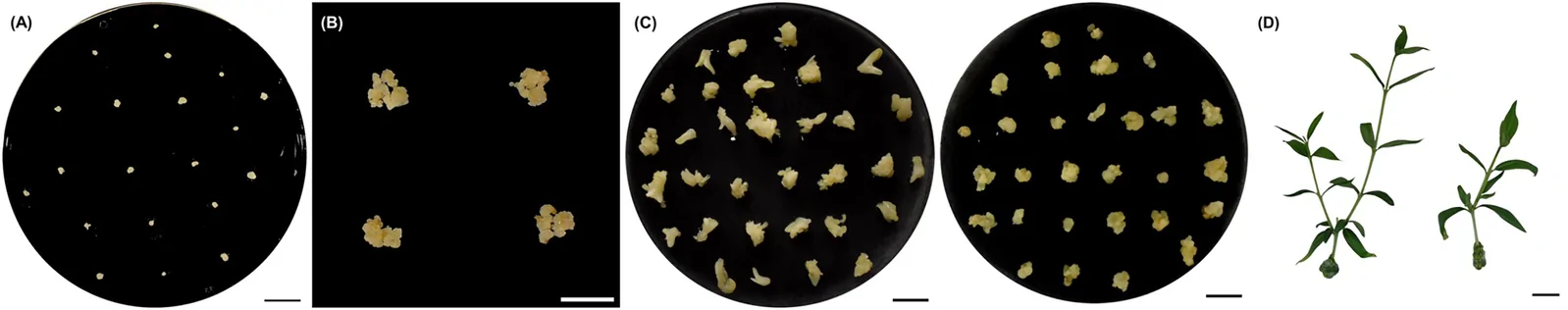
Regeneration of tetraploid olive plants from somatic embryos. **(A)** Globular somatic embryos cultured after colchicine treatment on ECO medium. **(B)** Subdivision of embryogenic callus developed in surviving line 31 for flow cytometric analysis. **(C)** Embryo maturation of diploid P1(left) and tetraploid 31(right) on hormone-free ECO medium supplemented with activated charcoal. **(D)** *In vitro* shoot multiplication of diploid P1 (left) and tetraploid 31(right) after 6 weeks of culture on RP medium. Scale bar = 1 cm.

To corroborate the ploidy levels determined by flow cytometry in leaf tissue, chromosome counting was performed on root-tip cells as a direct validation method. The diploid olive line P1 showed 46 chromosomes (2n = 46) (**Figure 3A**), whereas the tetraploid line 33 exhibited 92 chromosomes (4n = 92) (**Figure 3B**), as well as the other lines under study, 31 and 44 (**Supplementary Figure 1**). The strong alignment between both independent methods, conducted on distinct tissue types, successfully verified the ploidy level of the lines and excluded any occurrence of mixoploidy or chimerism.

**Figure 3:**
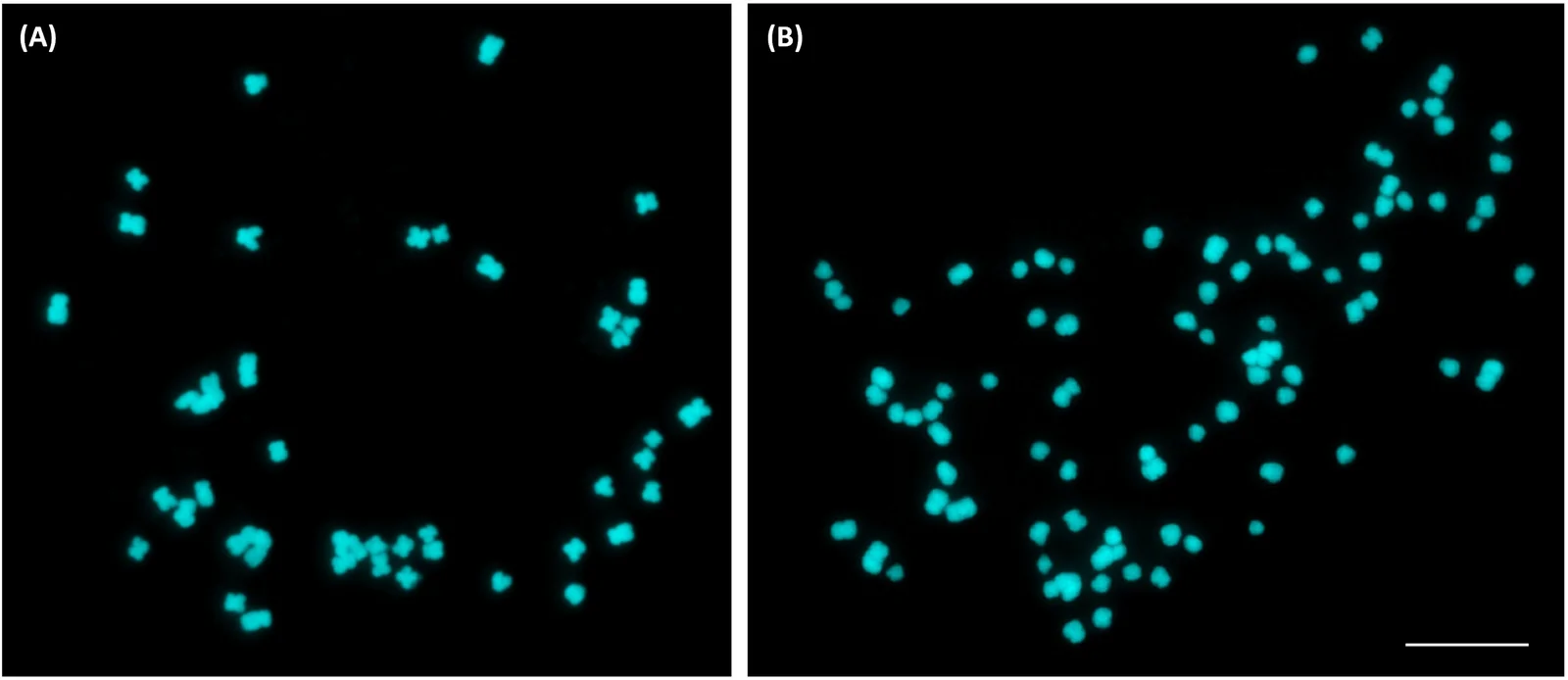
Mitotic metaphase chromosomes of olive in root-tip cells. **(A)** Representative image of diploid line P1 showing 46 chromosomes (2n= 2x = 46). **(B)** Representative image of a tetraploid line showing 92 chromosomes (2n = 4x = 92). The image shown corresponds to line 33. Chromosomes were counterstained with DAPI. Scale bar = 10 µm.

### 3.3 Micropropagation of tetraploid plants

Ploidy-dependent growth differences were initially detected during the *in vitro* multiplication phase. After eight weeks of culture, the number and length of axillary shoots were significantly lower in all tetraploid lines compared to the diploid control P1 (**Figures 4A, B**). No significant differences in these parameters were observed among the three tetraploid genotypes. In contrast, rooting capacity was not affected by genome duplication in the obtained lines. All evaluated tetraploid plants exhibited rooting responses near 100%, comparable to those of the diploid control, under the established culture conditions.

**Figure 4:**
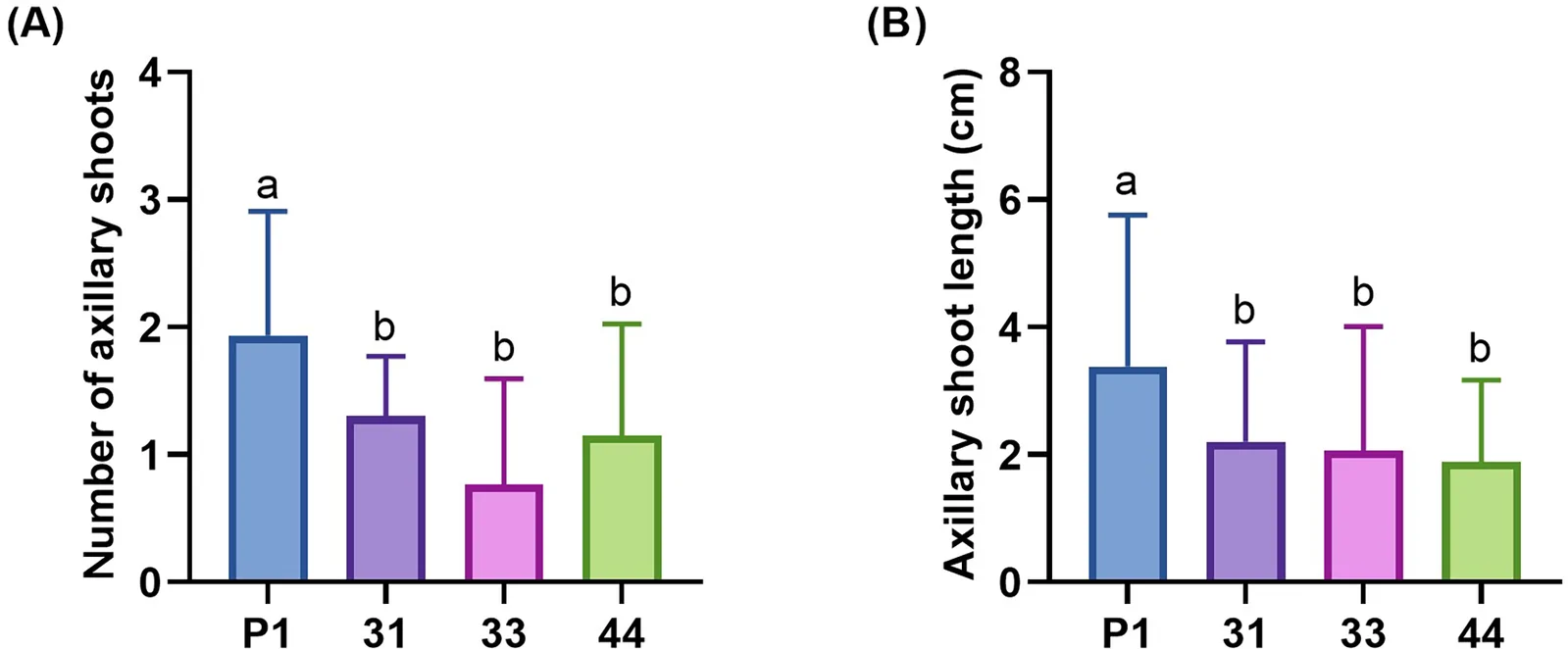
*In vitro* performance of the olive tetraploid lines during the multiplication phase. Nodal sections of diploid (P1) and tetraploid (31, 33, and 44) lines were cultured in olive micropropagation medium, and the number **(A)** and length **(B)** of axillary shoots were measured after 8 weeks for three subcultures. Values represent mean ± SD. Different letters indicate significant differences according to Welch’s ANOVA followed by Games-Howell *post hoc* test (p< 0.05).

### 3.4 Morphological and anatomical characterization of tetraploid plants

Distinct phenotypic divergences emerged once the plants were established under greenhouse conditions. In six-month-old plants, plant height was reduced in all tetraploid genotypes, with decreases ranging from 39.8% to 50.0% compared to the diploid control P1 (**Figure 5A**); an allometric relationship between plant height and stem diameter was analyzed to determine if the architecture of tetraploid plants had been affected (**Table 2**). All genotypes shared a common scaling slope (β = 1.93; P > 0.05 for slope heterogeneity; **Supplementary Figure 2**), indicating a conserved growth trajectory across lines. However, significant differences were detected in the elevation (intercept) of the regression lines among genotypes (**Table 2**). The four tetraploid lines exhibited lower elevations than the diploid control, demonstrating that tetraploid plants are significantly stockier relative to their height (**Table 2**; **Supplementary Figure 2**). On the other hand, no significant differences were observed in internode length across genotypes (**Table 3**).

**Figure 5:**
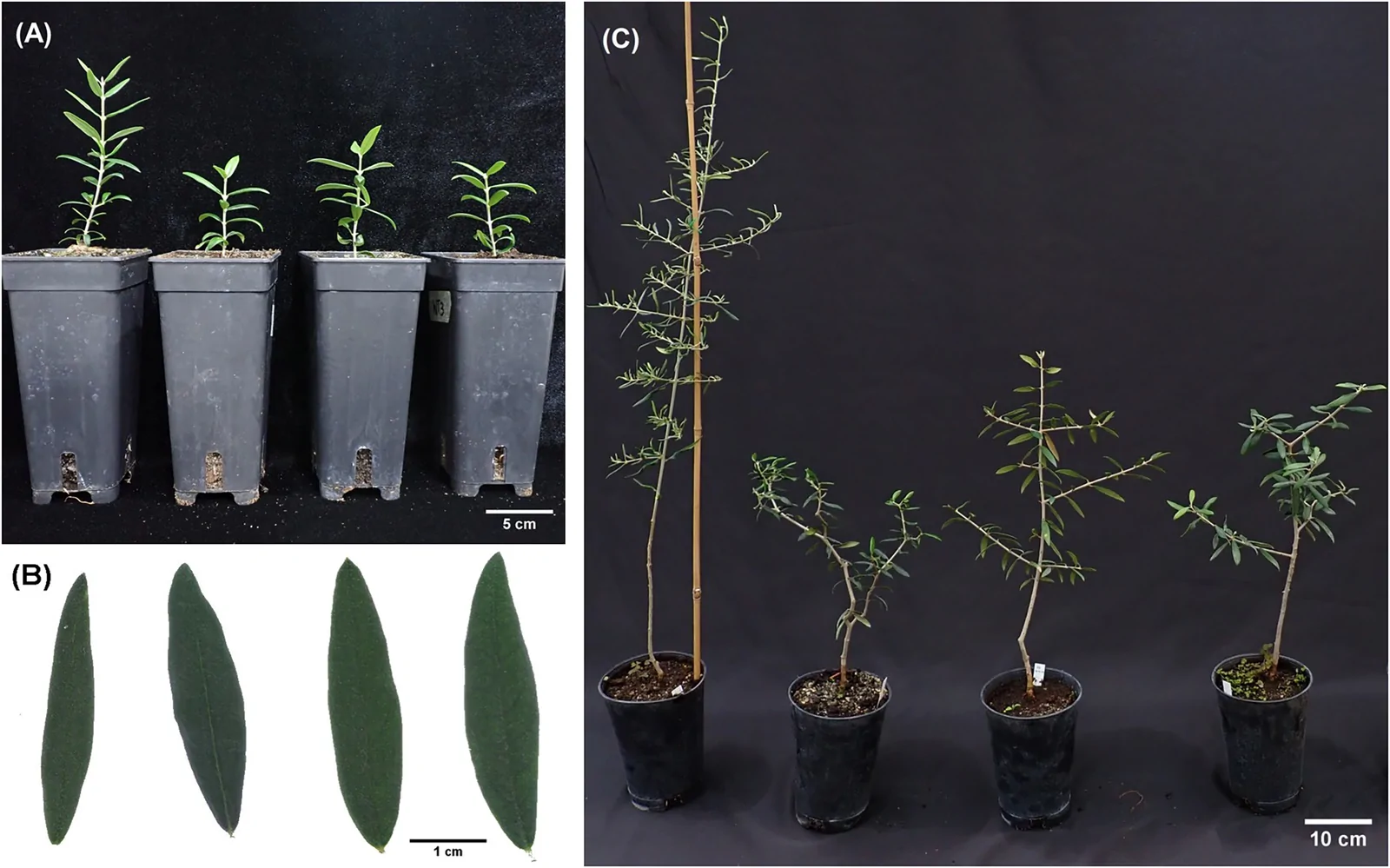
Morphological comparison between diploid and tetraploid olive plants grown under greenhouse conditions. **(A)** Six-month-old ex vitro plants of diploid line P1 (2n) and tetraploid lines 31, 33, 44 (4n), arranged from left to right. **(B)** Leaves corresponding to the plants shown in **(A)**, with the same left-to-right order. **(C)** Three-year-old plants of diploid line P1 and tetraploid lines 31, 32, 33, and 44, arranged from left to right.

**Table 2:** Morphological parameters and standardized major axis (SMA) allometric regressions relating plant height and stem diameter across diploid control (P1) and tetraploid (31, 33, 44) olive lines.

Genotype	Plant height (cm)	Stem diameter (mm)	SMA Elevation (α)	R^2^
P1	12.8 ± 0.59	2.07 ± 0.06	0.494^a^	0.47
31	6.45 ± 0.47	2.06 ± 0.10	0.195^b^	0.33
33	7.75 ± 0.48	2.03 ± 0.08	0.239^b^	0.68
44	7.02 ± 0.59	2.07 ± 0.06	0.240^b^	0.34

Values for height and diameter represent means ± SE (n = 15 plants per genotype).

Different letters within the “SMA Elevation” column indicate statistically significant differences between genotypes based on pairwise *post-hoc* tests adjusted for multiple comparisons (P< 0.05).

Allometric parameters were estimated from base-10 log-transformed data. A common slope (β = 1.93) was fitted across all lines as slope homogeneity was supported (P > 0.05).

**Table 3:** Morphological and anatomical parameters of six-month-old plants of diploid (P1) and tetraploid (31, 33, 44) olive lines growing under greenhouse conditions.

	Diploid	Tetraploid		
	P1	31	33	44
Internode length (cm)	1.0 ± 0.1^a^	1.1 ± 0.2^a^	1.0 ± 0.1^a^	1.0 ± 0.1^a^
Leaf area (mm^2^)	166.3 ± 28.3^c^	238.1 ± 46.9^a^	226.9 ± 44.7^ab^	202,6 ± 51.0^b^
Leaf length (mm)	34.5 ± 3.4^bc^	37.7 ± 3.2^a^	37.5 ± 4.0^ab^	34.3 ± 6.2^c^
Leaf width (mm)	6.5 ± 0.7^c^	8.9 ± 1.3^a^	8.2 ± 0.9^b^	7.9 ± 1.2^b^

Values represent mean ± SD.

Different letters indicate significant differences in the same row, according to one-way ANOVA followed by Tukey’s HDS test (p< 0.05).

Feret diameters were used as morphological indicators for leaf length and width.

Leaf morphology provided clear indicators of the gigas effect at this stage (**Figure 5B**). Across all tetraploid lines, leaves exhibited a significant increase in leaf area and leaf width compared with the diploid control, consistent with a predominantly lateral expansion of the lamina, although the magnitude of these changes varied among lines. Leaf area increased significantly by 22% to 43% compared with P1, with line 31 exhibiting the highest expansion (238.1 ± 46.9 mm^2^ vs. 166.3 ± 28.3 mm^2^ in P1; **Table 3**). Consistently, leaf width was enhanced by 21% to 37% in all 4n lines, whereas leaf length was less affected, increasing significantly only in line 31 (37.7 ± 3.2 mm) while remaining statistically comparable to P1 in lines 33 and 44 (**Table 3**).

Significant alterations were also observed in stomatal traits (**Figure 6**). Stomatal density was consistently reduced in all tetraploid plants compared to the diploid control, with decreases of approximately 23% in line 31, 40% in line 33 and 44% in line 44 (**Figure 6A**). Conversely, stomata size was significantly enhanced; all tetraploid lines exhibited significantly longer stomata than P1, with average lengths increasing from 19.3 µm in the diploid control to a range of 23.4 – 25.8 µm across the tetraploid lines (**Figure 6B**). Representative images of the abaxial leaf surface illustrating these differences in stomata size and distribution are shown in **Figures 6C, D**.

**Figure 6:**
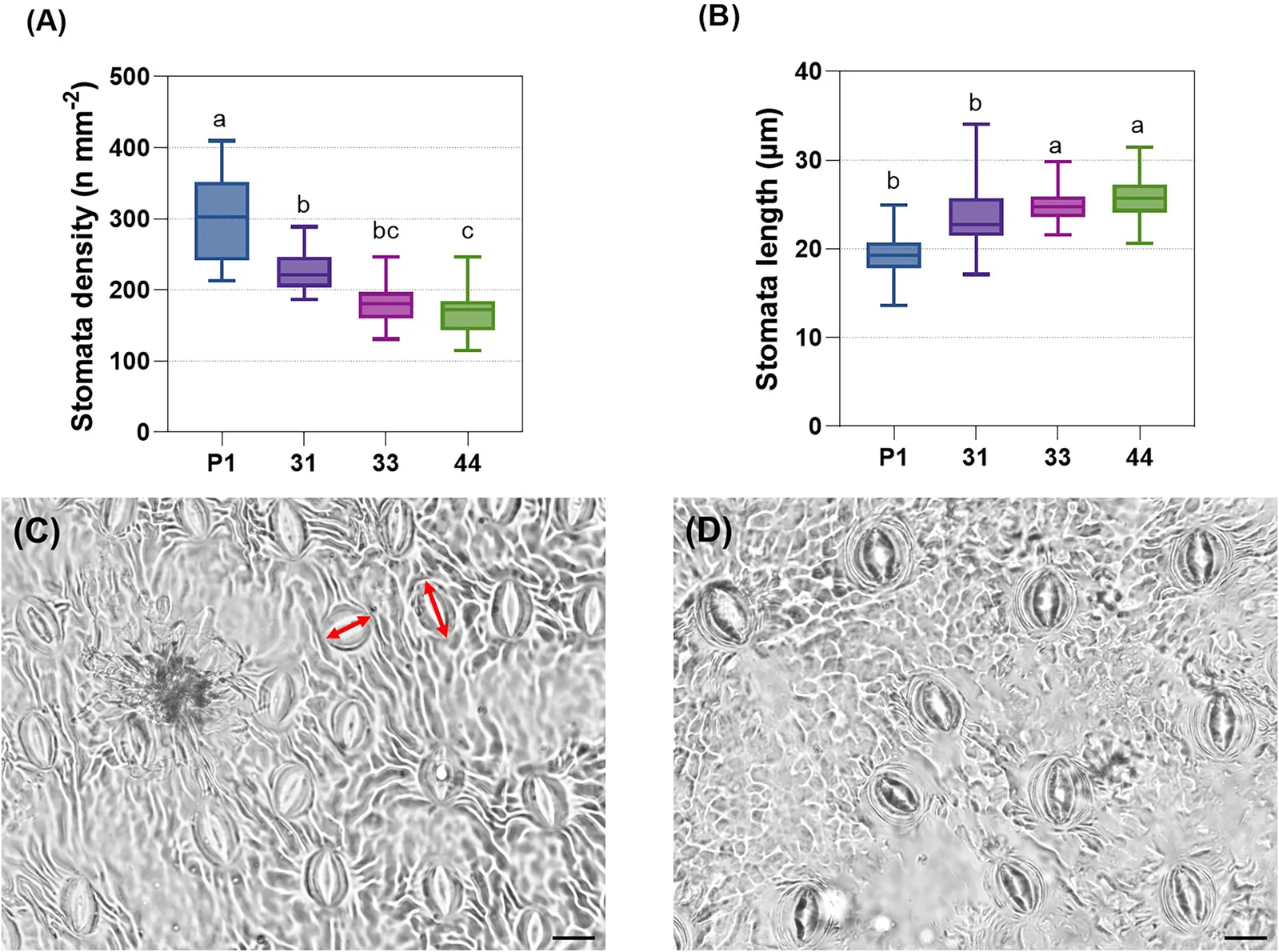
Comparison of stomata density and size between diploid and tetraploid olive plants. Boxplots showing stomata density **(A)** and stomatal length **(B)** of lines P1, 31, 33, and 44. Abaxial leaf surface of control diploid plant (line P1) **(C)** showing stomatal length measurement highlighted with red arrows, and tetraploid plant (line 44) **(D)**. Scale bar = 20 µm.

The growth divergence was further accentuated over consecutive years; by the third year, tetraploid lines reached less than half the plant height of the P1 control (**Figure 5C**). Anatomical studies conducted on these established plants confirmed that tetraploid leaves were significantly thicker, with an average increase in leaf lamina thickness of 39% (**Table 4**).

**Table 4:** Comparison of leaf anatomical structure of diploid (P1) and tetraploid (31, 33, 44) olive lines.

Leaf thickness (different cell layers)	Diploid	Tetraploid		
	P1	31	33	44
Total leaf thickness (mm)	0.359 ± 0.051^b^	0.494 ± 0.034^a^	0.496 ± 0.037^a^	0.503 ± 0.027^a^
Cuticle (µm)	4.13 ± 0.03^a^	3.52 ± 0.36^a^	4.96 ± 0.73^a^	4.69 ± 0.82^a^
Adaxial epidermis (µm)	16.1 ± 1.9^b^	21.3 ± 2.4^a^	15.6 ± 0.5^b^	17.2 ± 1.9^ab^
Palisade parenchyma (µm)	64.1 ± 6.0^b^	114.9 ± 5.3^a^	115.1 ± 11.3^a^	117.9 ± 16.4^a^
Spongy parenchyma (µm)	240.8 ± 29.2^a^	257.2 ± 3.6^a^	261.8 ± 34.0^a^	256.1 ± 20.3^a^
Abaxial epidermis (µm)	11.6 ± 0.2^a^	12.8 ± 0.8 ^a^	12.8 ± 0.8^a^	12.8 ± 0.4^a^
Cell area				
Adaxial epidermis (µm^2^)	163.6 ± 14.5^c^	455.9 ± 14.2^a^	354.3 ± 18.0^b^	335.8 ± 8.2^b^
Palisade parenchyma (µm^2^)	292.6 ± 24.9^c^	897.8 ± 92.2^a^	777.9 ± 79.0^ab^	692.6 ± 86.2^b^
Spongy parenchyma (µm^2^)	365.2 ± 39.7^c^	781.0 ± 35.8^a^	632.0 ± 45.3^b^	704.2 ± 69.0^ab^
Abaxial epidermis (µm^2^)	141.4 ± 4.3^a^	190.4 ± 43.2 ^a^	189.5 ± 14.6^a^	213.1 ± 61.9^a^

Values represent mean ± SD.

Different letters indicate significant differences in the same row, according to one-way ANOVA followed by Tukey’s HDS test (p< 0.05).

Data include thickness of the different leaf cell layers as well as area of the different cell types.

Histological analysis (**Figure 7**) further resolved these differences at the tissue level. Tetraploid lines showed significantly greater palisade parenchyma thickness than the diploid control P1, with values ranging from 114.9 to 117.9 µm compared with 64.1 ± 6.0 µm in P1 (**Figures 7A–D**; **Table 4**). Adaxial epidermis thickness was also significantly greater in line 31 (21.3 ± 2.4 µm) than in P1 (16.1 ± 1.9 µm) and line 33 (15.6 ± 0.5 µm), whereas line 44 showed an intermediate value and did not differ significantly from the other genotypes (**Table 4**). In contrast, no significant differences were detected among genotypes in cuticle, spongy parenchyma or abaxial epidermis thickness (**Table 4**).

**Figure 7:**
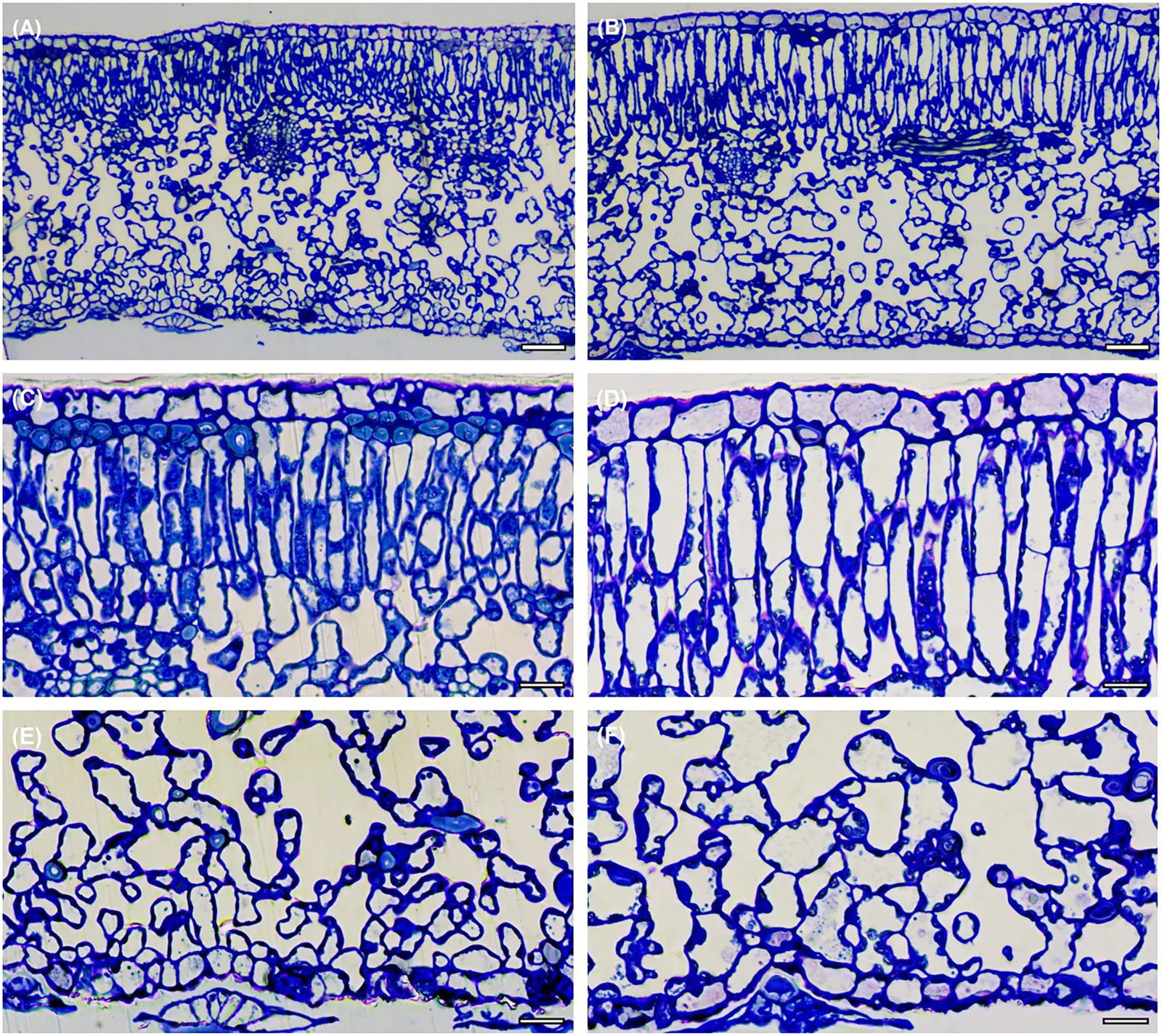
Anatomy of the leaf lamina in diploid and tetraploid olive plants. **(A)** Cross section of an olive leaf control diploid plant (line P1). **(B)** Cross section of a leaf from the tetraploid line 31. **(C, D)** Detailed images of the abaxial epidermis and palisade parenchyma of diploid line P1 **(C)** and tetraploid line 31 **(D)**. **(E, F)** Detailed images of the spongy parenchyma, abaxial epidermis of diploid plant P1 **(E)** and tetraploid plant 31 **(F)**. Scale bar **(A, B)** = 50 µm, Scale bar **(C–F)** = 20 µm.

Finally, cell area measurements revealed a pronounced enlargement in the tetraploid lines for the adaxial epidermis, palisade parenchyma, and spongy parenchyma. In the adaxial epidermis, all tetraploid lines exhibited higher values than the control P1, with line 31 showed the highest one (455.9 ± 14.2 µm^2^). Similarly, in palisade and spongy parenchyma, cell area was significantly greater in tetraploid lines in comparison to the control, again with 31 giving the highest value. No significant differences were detected among genotypes in abaxial epidermal cell area (**Table 4**).

### 3.5 Physiological characterization

Photosynthetic performance was assessed using PAM fluorometry. Under greenhouse conditions, tetraploid lines consistently achieved higher ETR values than P1, although the difference was only significant in the case of line 44 (**Table 5**). By contrast, the apparent quantum efficiency (α) was similar in all genotypes. The Fv/Fm rate also remained stable across genotypes, although line 44 showed a slight but significant reduction compared to P1 and line 31. As regards the stomatal conductance, values in tetraploid lines remained comparable to those of the diploid control, although significant differences were observed among tetraploid lines, with line 31 showing the highest gs value and lines 33 and 44 exhibiting lower conductance.

**Table 5:** Photosynthetic performance parameters in diploid (P1) and tetraploid (31, 33, 44 lines) olive plants.

	Diploid	Tetraploid		
	P1	31	33	44
Apparent quantum efficiency (α)	0.137 ± 0.014^a^	0.134 ± 0.007^a^	0.132 ± 0.017^a^	0.133 ± 0.010^a^
Electron transfer rate (ETR) (μmol electrons m^-2^s^-1^)	86.3 ± 7.6^b^	109.4 ± 12.7^ab^	110.3 ± 18.6^ab^	113.4 ± 15.4^a^
F_v_/F_m_	0.751 ± 0.031^a^	0.741 ± 0.025^a^	0.737 ± 0.026^ab^	0.670 ± 0.063^b^
gs (mmol m^−2^ s^−^)	184.9 ± 24.4^ab^	217.6 ± 27.7^a^	164.6 ± 21.9^b^	165.6 ± 35.8^b^

Values represent mean ± SD.

Different letter indicates significant differences in the same row, according to one-way ANOVA followed by Tukey’s HDS test (p< 0.05).

Apparent quantum efficiency (α), electron transfer rate (ETR) at saturating irradiance, and Fv/Fm rate were measured with a fluorescence Junior PAM, while stomatic conductance was measured with a leaf porometer.

Leaf water status was further assessed under high-RH and low-RH conditions (**Figure 8**). Under high-RH (**Figure 8A**), no significant differences were detected among lines, with mean RWC values ranging from 70.50% (P1) to 77.23% (line 33). However, under low-RH (**Figure 8B**), tetraploid lines maintained significantly higher RWC values than control P1. Leaves from line 44 showed the highest stability in water status, maintaining a mean RWC of 68.30% under low-RH, corresponding to the smallest RWC reduction among all genotypes (-7.30%). Despite these differences in the RWC, the amount of WLR (water loss by leaf area unit) did not differ between tetraploids and control P1, in both conditions (**Figures 8C, D**). Line 33 showed the lowest WLR, whereas line 31 exhibited the highest values; significant differences were observed only between these two lines, and no other pairwise comparisons were statistically significant.

**Figure 8:**
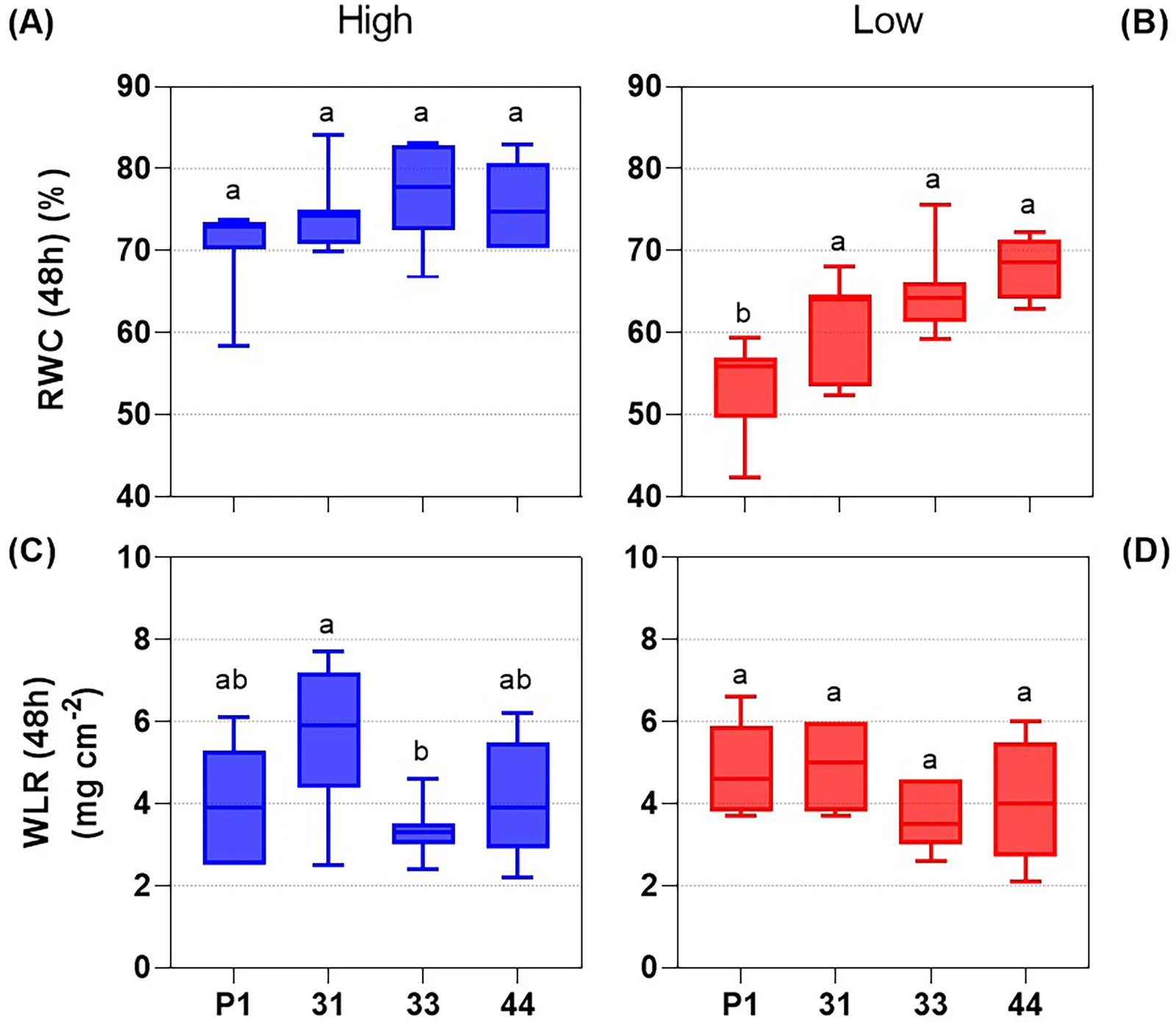
Physiological leaf traits responses under high (blue) and low (red) water availability conditions in diploid P1 and tetraploid 31, 33, and 44 lines after 48 h treatment. Relative water content (RWC) under high **(A)** and low **(B)** RH treatment expressed in percentage. Water loss rate (WLR) under high **(C)** and low **(D)** RH treatment expressed in mg cm^-2^. Boxes represent the interquartile range, the central line indicates the median, and different letters above the boxes indicate statistically significant differences (p< 0.05) among lines within each treatment according to Sidak’s multiple comparisons test.

Regarding LMA (leaf mass per area), all tetraploid lines exhibited higher values than the control P1 (**Figure 9**), with line 44 outperforming all of them.

**Figure 9:**
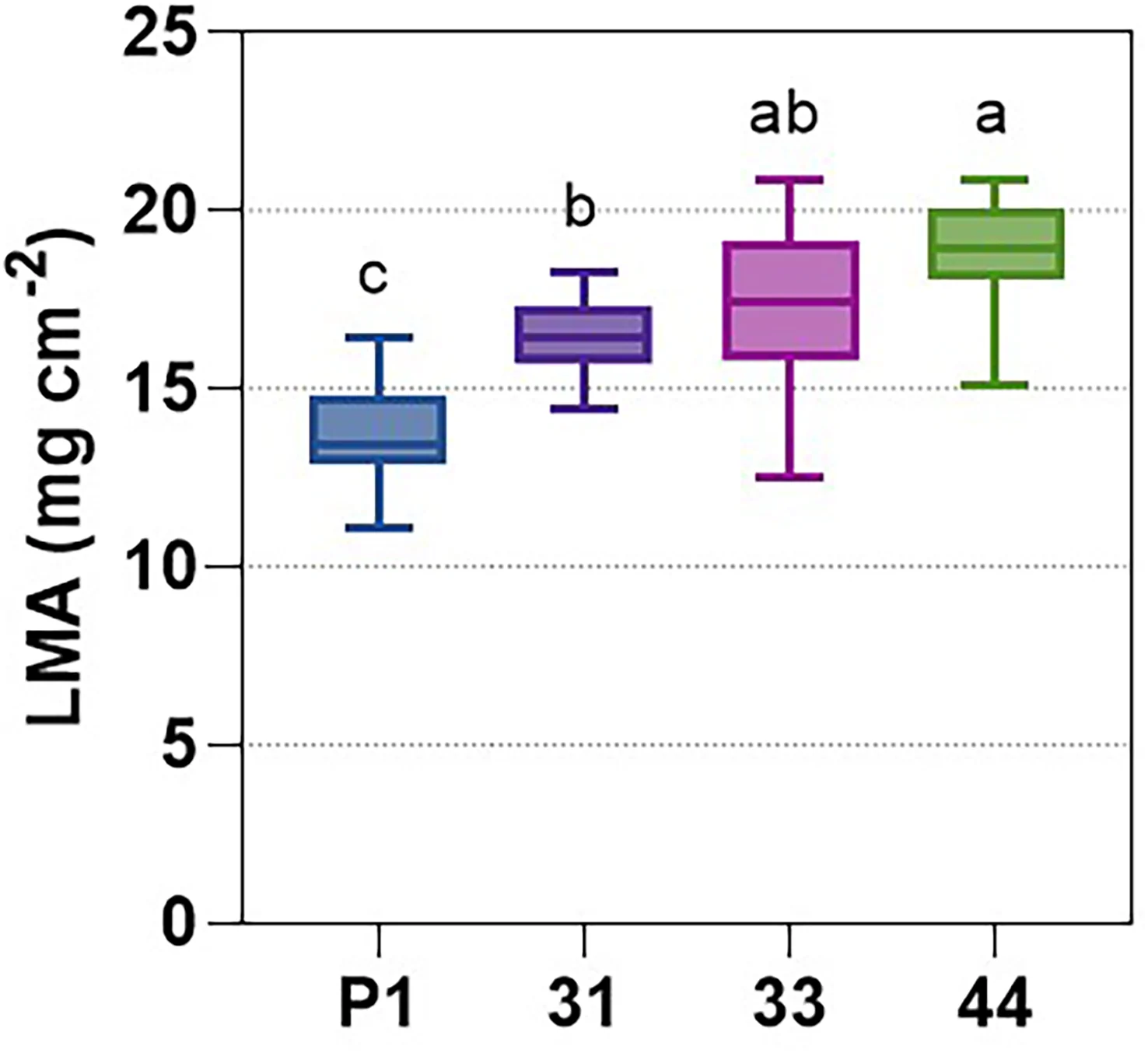
Comparison of leaf mass per area (LMA) among diploid P1 and tetraploid 31, 33, and 44 lines in mg cm^−2^. Letters above the boxes indicate statistically significant differences (p< 0.05) according to Tukey’s multiple comparisons test. The center line in each box represents the median, while the whiskers indicate the full range of the data.

## 4 Discussion

### 4.1 Induction of polyploidy in olive embryogenic callus

Colchicine is widely used to induce polyploidy in plants. The balance between survival rate and induction efficiency is critical, as it requires conditions close to the LD_50_ threshold to maximize chromosome doubling frequency (Eng and Ho, 2019). In general, low colchicine concentrations or short exposure times are insufficient to induce polyploidy efficiently (Gu et al., 2005; Widoretno, 2016). In the present study, treatment with 0.1% colchicine combined with short exposure times such as 1 day was ineffective for polyploid induction. In contrast, extending the treatment to 6 days increased the proportion of polyploid embryos up to 12.5%; however, this response was accompanied by a drastic reduction in explant survival (6.5%), which severely limits the practical applicability of this approach. This pattern reflects a widely described phenomenon (Song et al., 1997; Wu and Mooney, 2002; Lin et al., 2023) in which dose and exposure time affect cytotoxicity as a result of colchicine interference with microtubule polymerization and cell cycle progression, compromising cell viability (Caperta et al., 2006). In the case of ornamental ginger (*Hedychium muluense*), embryogenic calli were exposed to colchicine at different concentrations (0, 2.5, 5 and 10 mM) during 1, 2 and 3 days. A clear cytotoxic threshold was identified during polyploidy induction, where concentrations above 5 mM colchicine severely compromised the system, although lower doses (0–5 mM) caused minimal variation in embryo yield (Sakhanokho et al., 2009). Consistently, our results in olive indicate that 2–3 days treatments with 0.1% colchicine achieve relevant induction levels (2.82 – 9.32%) while maintaining regeneration-compatible viability.

A few months after the ploidy assessment, a second flow cytometry analysis was performed to confirm the maintenance of the tetraploid nature in each line. Some of them maintained tetraploidy homogeneously, while others showed mixed profiles, containing both 4n and 2n callus sectors. These findings suggest that, within a single embryogenic callus, some regions may be fully tetraploid while others remain diploid, and that during proliferation, the diploid sectors may compete and gradually dominate the tetraploid regions. This phenomenon, named Population effect (Zhang et al., 2007), was observed in callus cultures of ‘Anliucheng’ sweet orange (*Citrus sinensis*) derived from abortive ovules. They showed that when polyploid cells were present at low frequency (11%), plant regeneration yielded only diploid individuals, whereas an increase in polyploid cell density to 59% shifted the outcome towards tetraploids, with approximately 54.5% of regenerated plants being non-chimeric tetraploids.

The observation that diploid sectors may progressively outcompete tetraploid ones during proliferation highlights the importance of repeated flow cytometry screening to ensure the genetic stability of regenerated material. This interpretation is consistent with classical observations in colchicine-treated rose meristems, where sectorial cytochimeras arise, and diploid cell layers tend to overgrow polyploid sectors over time (Semeniuk and Arisumi, 1968). This is known as diplontic selection, a process in which diploid cells with higher division rates outcompete polyploid cells (Klekowski, 2003). In our study, when sectorial chimerism was observed, the lines were discarded.

The successful establishment of several stable tetraploid lines demonstrates the suitability of small globular SEs for avoiding permanent chimerism in olive. According to Eng and Ho (2019), the use of small-sized explants enhances colchicine penetration into target cells, improving chromosome doubling efficiency. Moreover, regeneration from a reduced number of cells, potentially of unicellular origin, minimizes mixoploidy, as reported by Regalado et al. (2017) and Predieri (2001). Tetraploids have been successfully obtained from embryogenic callus in *Citrus* (Gmitter et al., 1991; Wu and Mooney, 2002), while in *Vitis*, either embryogenic callus (Yang et al., 2006) or suspension cultures (Acanda et al., 2015) have been used. This strategy overcomes limitations associated with other explants, such as shoot tips or nodal segments, in which multiple histological layers must be affected simultaneously (Touchell et al., 2020); in these cases, higher rates of mixoploidy have been reported (Cai et al., 2015).

Rugini et al. (1996) reported the regeneration of olive plants through micropropagation, via axillary bud stimulation, of previously identified mixoploid mutants; shoots with different proportions of euploid (2n, 4n) and aneuploid cells were selected during successive subcultures under *in vitro* conditions. Their study showed that culture conditions can promote euploid selection, although aneuploid cells may persist in some regenerated shoots, highlighting the cytogenetic instability associated with these regeneration routes in olive. In our case, the use of somatic embryos overcame these problems, allowing the obtainment of non-chimeric tetraploid lines.

### 4.2 *In vitro* growth, acclimatization, and vegetative architecture of tetraploid olive plants

Genome duplication induced profound structural and developmental changes that were evident both during *in vitro* shoot multiplication and after ex vitro acclimatization. During *in vitro* proliferation tetraploid olive lines exhibited reduced elongation, shorter internodes, and a noticeably more compact growth habit compared to the diploid control. These observations align with reports in other woody and horticultural autopolyploids, such as *Ribes nigrum* (Podwyszyńska and Pluta, 2019) or *Gerbera jamesonii* (Mahanta et al., 2023), where chromosome doubling routinely restricts shoot elongation and proliferation dynamics *in vitro*.

Several studies reported that differences observed under *in vitro* conditions were maintained in acclimatized plants (Carmona-Martín et al., 2014; Regalado et al., 2017). Genome duplication has been widely associated with morphological alterations and the so-called gigas effect (Dhooghe et al., 2011). Usually, autotetraploid lines from woody species such as *Citrus* (Allario et al., 2011; Guerra et al., 2014), *Malus* (Ma et al., 2016), and *Broussonetia papyrifera* (Lin et al., 2023), showed reduced vegetative growth and a tendency towards more compact architectures compared with diploid controls. Similarly, in ornamental and model herbaceous systems such as *Petunia* (Van Iersel and Nemali, 2004; Regalado et al., 2017), *Platanus* (Liu et al., 2007), *Spathiphyllum* (Van Laere et al., 2010), and *Glycine* (Zhai et al., 2025), reduced vegetative vigor and altered plant architecture have been consistently reported. In the present study, the reduction in plant height observed in all tetraploid lines suggests a decreased elongation capacity associated with genome duplication.

The allometric analysis performed in this work revealed that although tetraploid plants were significantly shorter, they maintained stem diameters comparable to those of the diploid control, resulting in a more robust growth habit. This finding is particularly relevant when interpreting the effects of polyploidization on plant architecture. Pannelli et al. (1990, 1992) previously observed that tetraploid olive mutants derived from gamma irradiation of ‘Frantoio’ and ‘Leccino’ exhibited reduced internode length and a more compact growth form, although their study did not perform quantitative allometric analyses. Afterwards, Paoletti and Rosati (2025) characterized morphological differences in one-year-old shoots derived from those tetraploid and diploid ‘Leccino’ plants, reporting larger diameter stems but unchanged internode lengths in tetraploids. Our results demonstrate that the compactness observed in tetraploid olive is not merely a consequence of reduced height but reflects an altered allometric relationship between height and diameter, indicating a fundamental change in growth allocation patterns following genome duplication, which confirms Paoletti and Rosati (2025) analysis at shoot level in ‘Leccino’.

Leaf morphology is also one of the most informative traits associated with genome duplication. In general, tetraploid olive lines developed wider leaves than the diploid control, whereas changes in leaf length were less consistent among lines. Similar traits have previously been reported in the olive itself (Pannelli et al., 1990; 1992; Paoletti and Rosati, 2025), *Vaccinium duclouxii* (Lei et al., 2023), *Luculia pinceana* (Wang et al., 2025a), *Buddleja madagascarensis* × *B. crispa* (Dunn and Lindstrom, 2007), *Pogostemon cablin* (Widoretno, 2016), and *Pyrus communis* (Sun et al., 2015). In our tetraploid plants, leaf width appeared to be more consistently affected than leaf length, which suggests that polyploidization may preferentially promote lateral expansion of the lamina rather than uniform longitudinal growth. The more moderate increase in leaf area observed in line 44 compared with lines 31 and 33 further supports the existence of genotype-dependent responses to polyploidization, as all three lines share the same ploidy level but differ in the magnitude of their morphological changes. Since all tetraploid lines originated from the same embryogenic genotype (P1), the phenotypic differences observed among them are unlikely to reflect pre-existing genetic variation; instead, they may result from outcomes independent of genome duplication, as well as from epigenetic changes leading to alterations in gene expression regulation (Li et al., 2011) or from somaclonal variation associated with *in vitro* culture.

### 4.3 Anatomical modifications underlying the gigas effect

Studies addressing anatomical changes in polyploid plants have most commonly focused on tissue-level structural traits, particularly leaf thickness, as reported in tetraploid cassava variety ‘Xinxuan 048’ (Zhou et al., 2017), *Lilium* FO hybrids (Cao et al., 2018), *Arabidopsis thaliana* (Corneillie et al., 2019), banana (*Musa* spp.) (Madail et al., 2022), *Borago officinalis* (Šenkyřík et al., 2025), or *Populus davidiana* (Wang et al., 2025b). Increased leaf thickness observed in tetraploid genotypes is consistent with the classical gigas effect associated with polyploidy. This phenomenon has been widely discussed in the context of genome size scaling, which links increases in nuclear DNA content with enhanced cell size and coordinated changes in organ morphology (Knight and Beaulieu, 2008). Within this framework, the present study combines tissue level measurements with quantitative analyses of cell area, providing a detailed mechanistic assessment of the structural basis of polyploid leaf phenotypes.

Tetraploid genotypes exhibited a pronounced and consistent increase in cell area across all leaf tissues, particularly in the mesophyll, where both palisade and spongy parenchyma cells showed substantial enlargement. In contrast, changes in tissue thickness were more limited, largely confined to the palisade parenchyma, while other tissues remained relatively stable. Pannelli et al. (1992) also observed larger cells in leaf tissues of tetraploid olive, although most tissue layers were affected, with the lamina, palisade, and spongy tissues thicker. This pattern indicates that the increase in leaf thickness is primarily driven by cell expansion rather than changes in tissue layering or cell proliferation. A similar response has been reported in tetraploid birch (*Betula pendula*), where increased leaf size was mainly attributed to enhanced cell expansion, whereas cell proliferation contributed little to organ enlargement (Zhang et al., 2022). In autotetraploid ‘Valencia’ sweet orange (*Citrus sinensis* (L.) Osb.) and ‘Femminello’ lemon (*Citrus limon* (L.) Burm. f.), genome duplication led to a substantial increase in mesophyll cell volume, accompanied by a lower number of cells per unit leaf area compared with diploids (Romero-Aranda et al., 1997). All these findings support the view that polyploid leaf enlargement is achieved through increased cell volume rather than increased cell number.

The enlargement of leaf cells observed in tetraploid genotypes is not restricted to internal tissues. Similar size-related responses are commonly reflected in epidermal structures, particularly stomata, which have been widely used as reliable indicators of ploidy level in *in vitro*-induced autopolyploid plants. Numerous studies have shown that genome duplication is typically associated with increased stomatal size and reduced stomatal density, making these traits simple and effective markers for indirect polyploid identification (Yang et al., 2006; Rego et al., 2011; Fonollá et al., 2023). This anatomical pattern was also observed in all our tetraploid lines. On the other hand, Caporali et al. (2014) also described an increase in cell size in reproductive organs of tetraploid ‘Leccino’ plants.

The variation observed among tetraploid lines in anatomical traits merits further consideration. Line 31 consistently exhibited the largest cell areas across most tissues, while line 44 showed more moderate increases, particularly in palisade parenchyma cell area. These differences may reflect the stochastic nature of the chromosome doubling event or subtle epigenetic modifications that occurred during the *in vitro* culture process. Such line-specific variation has been observed in other polyploid systems and underscores the importance of evaluating multiple independent tetraploid lines when assessing the effects of genome duplication (Osborn et al., 2003; Doyle and Coate, 2019).

### 4.4 Physiological implications: photosynthesis and water relations

PAM fluorometry revealed that tetraploid lines consistently achieved higher ETR values than P1, although the difference was only statistically significant in the case of line 44. This trend is consistent with previous reports in other polyploid systems, where increased mesophyll cell area and thicker leaves have been associated with enhanced photosynthetic capacity (Pannelli et al., 1990; Romero-Aranda et al., 1997; Cao et al., 2018). The absence of significant differences in α indicates that initial photochemical efficiency remained stable among genotypes, whereas Fv/Fm values remained within the normal range for healthy plants across all genotypes (>0.65), indicating that PSII functionality was largely maintained despite the physiological changes induced by polyploidy. However, line 44 showed a slight but significant reduction compared to P1, suggesting a minor genotype-specific effect on photochemical efficiency that does not appear to compromise overall photosynthetic performance. Similar responses have been reported in other polyploid species, such as *Lilium* FO hybrids and *Populus* genotypes (Liao et al., 2016; Cao et al., 2018).

The structural changes induced by polyploidization are expected to affect not only photosynthetic performance but also leaf water relations. Accordingly, water loss in detached leaves was evaluated as an integrative approach to assess drought tolerance, as it reflects stomatal regulation, cuticular transpiration, and leaf architectural traits (Hopper et al., 2014; Czyczyło-Mysza et al., 2018; López-Pozo et al., 2019). The results obtained in the tetraploid lines, particularly line 44, aligns with the selection criteria proposed by Razouk et al. (2022), who identified water loss in detached leaves as a primary phenotypic marker for drought tolerance in olive. In their study, WLR showed an extremely strong correlation with yield stability and water use efficiency. Accordingly, the ability of line 44 to maintain a higher RWC and regulate WLR under desiccation conditions could reflect higher yield potential under stress.

The performance of line 44 resembles that of highly drought-tolerant cultivars such as ‘Lechin de Sevilla’ and ‘Picholine Marocaine’, which are characterized by minimal water loss and a combination of small leaves and low stomatal density. This supports the observation that the 44% reduction in stomatal density in line 44, together with its higher LMA, reflects a more efficient anatomical strategy for limiting water loss. The reduction in stomatal density observed in tetraploid lines represents a trade-off between CO_2_ uptake and water loss. Under well-watered conditions, this anatomical modification did not compromise photosynthetic performance, as evidenced by the maintained or enhanced ETR values. However, under water-limiting conditions, the reduced stomatal density likely contributes to improved water conservation, as suggested by the higher RWC values maintained by tetraploid lines during desiccation. In this context, the higher LMA observed in line 44 suggests greater structural investment and may be associated with a more developed palisade parenchyma, as reported by Guerfel et al. (2009) in different olive cultivars, where drought-tolerant ‘Chemlali’ exhibited a more developed palisade parenchyma together with thicker epidermal and cuticular layers, contributing both to improved protection against dehydration and to the maintenance of photosynthetic activity under water-limited conditions. The positive correlation between LMA and drought tolerance has been widely documented in olive and other woody species, as higher LMA generally reflects increased leaf thickness, which contribute to reduced water loss and improved water use efficiency (Guerfel et al., 2009; Razouk et al., 2022).

The differential responses among tetraploid lines in water relations parameters further highlight the importance of evaluating multiple independent lines. Line 44 consistently outperformed the other tetraploid lines in terms of water retention capacity, suggesting that the specific outcomes of genome duplication, whether genetic, epigenetic, or stochastic, can result in lines with varying degrees of physiological improvement. This variability, rather than being a limitation, represents an opportunity for selecting superior genotypes for breeding programs aimed at developing drought-tolerant rootstocks or cultivars.

## 5 Conclusions

The use of embryogenic cultures enabled the efficient recovery of stable, non-chimeric tetraploid olive lines following colchicine treatment, representing the first report of tetraploid induction using SEs as explants in this species. Genome duplication induced a coordinated set of morphological and physiological modifications, including reduced plant height with stockier growth habit, wider and thicker leaves with increased cell size across tissues, reduced stomatal density, enhanced electron transport capacity, and improved water retention under low humidity conditions. Notably, line 44 exhibited the most favorable water relations, maintaining higher relative water content and leaf mass per area while showing the lowest water loss rate, suggesting its potential as a drought-tolerant genotype. These tetraploid lines provide a valuable genetic resource for investigating polyploidy effects in olive and may facilitate the future development of improved cultivars and rootstocks with enhanced resilience to environmental stress in the context of climate change and intensive production systems.

## Data Availability

The original contributions presented in the study are included in the article/**Supplementary Material**. Further inquiries can be directed to the corresponding author.
