# Supplementary material for: Stable tetraploid olive (Olea europaea L.) plants derived from embryogenic cultures show altered growth and improved water retention
Source: Front Plant Sci. 2026 Oct 13;17:1941427. doi: 10.3389/fpls.2026.1941427 (PMC13711110; doi:10.3389/fpls.2026.1941427)

Supplementary Material

Supplementary Figure 1. Mitotic metaphase chromosomes of tetraploid *O. europaea* L. lines showing 92 chromosomes (2n = 4x = 92). (A-B) Representative metaphase spreads of line 31. (C-D) Representative metaphase spreads of line 33. (E-F) Representative metaphase spreads of line 44. Chromosomes were counterstained with DAPI. Scale bar = 10 μm.

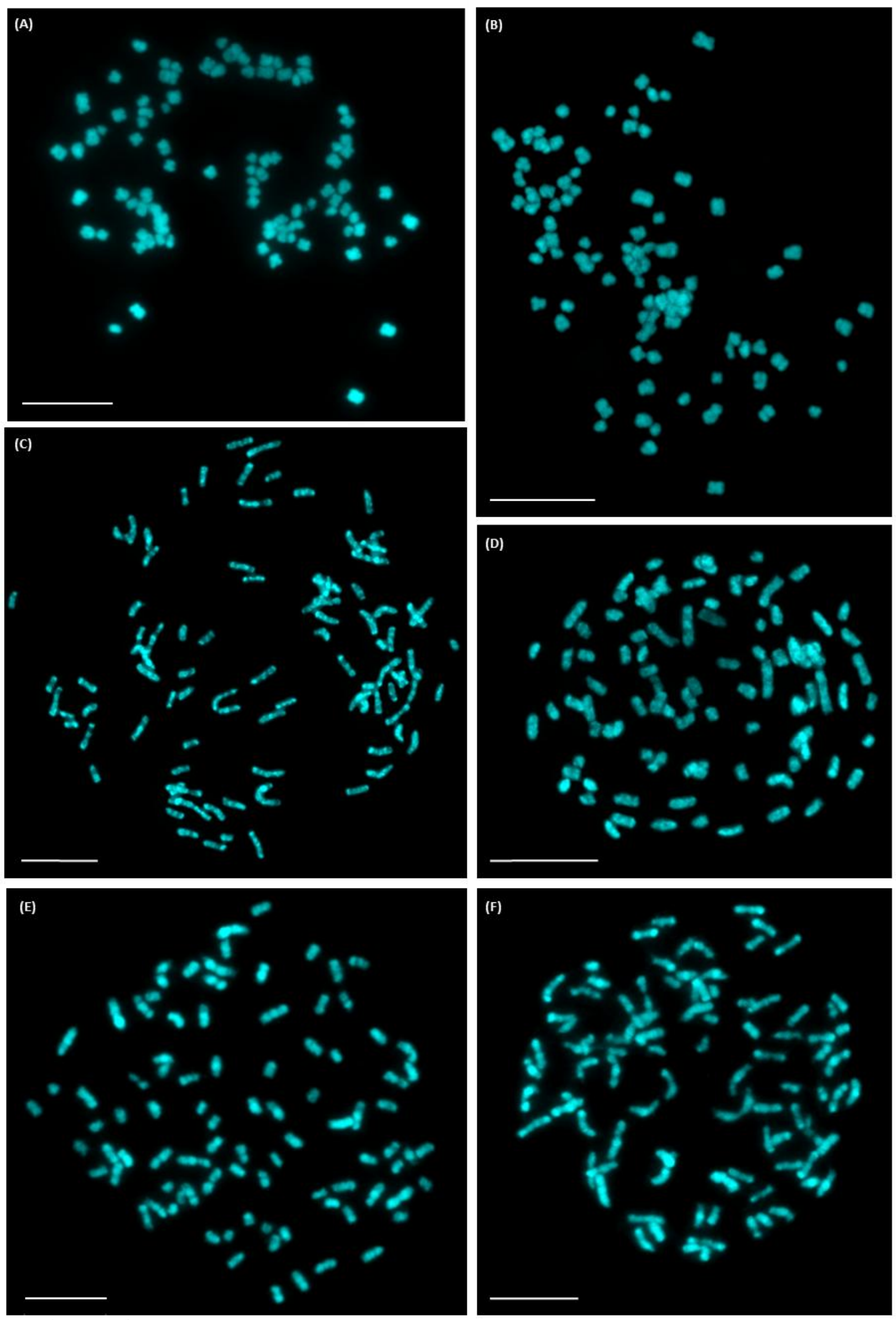

Supplementary Figure 2. Allometric relationship between stem diameter and plant height in diploid and tetraploid olive lines. Scatter plot displaying log-transformed individual data points and independent linear regression lines for the diploid control P1 and tetraploid lines 31, 33 and 44. Shaded areas represent 95% confidence intervals for each regression curve. The regression analysis illustrates a common growth trajectory among genotypes (homogeneous slopes) but a significant upward displacement in the elevations of the tetraploid lines, indicating a stockier growth habit relative to height.

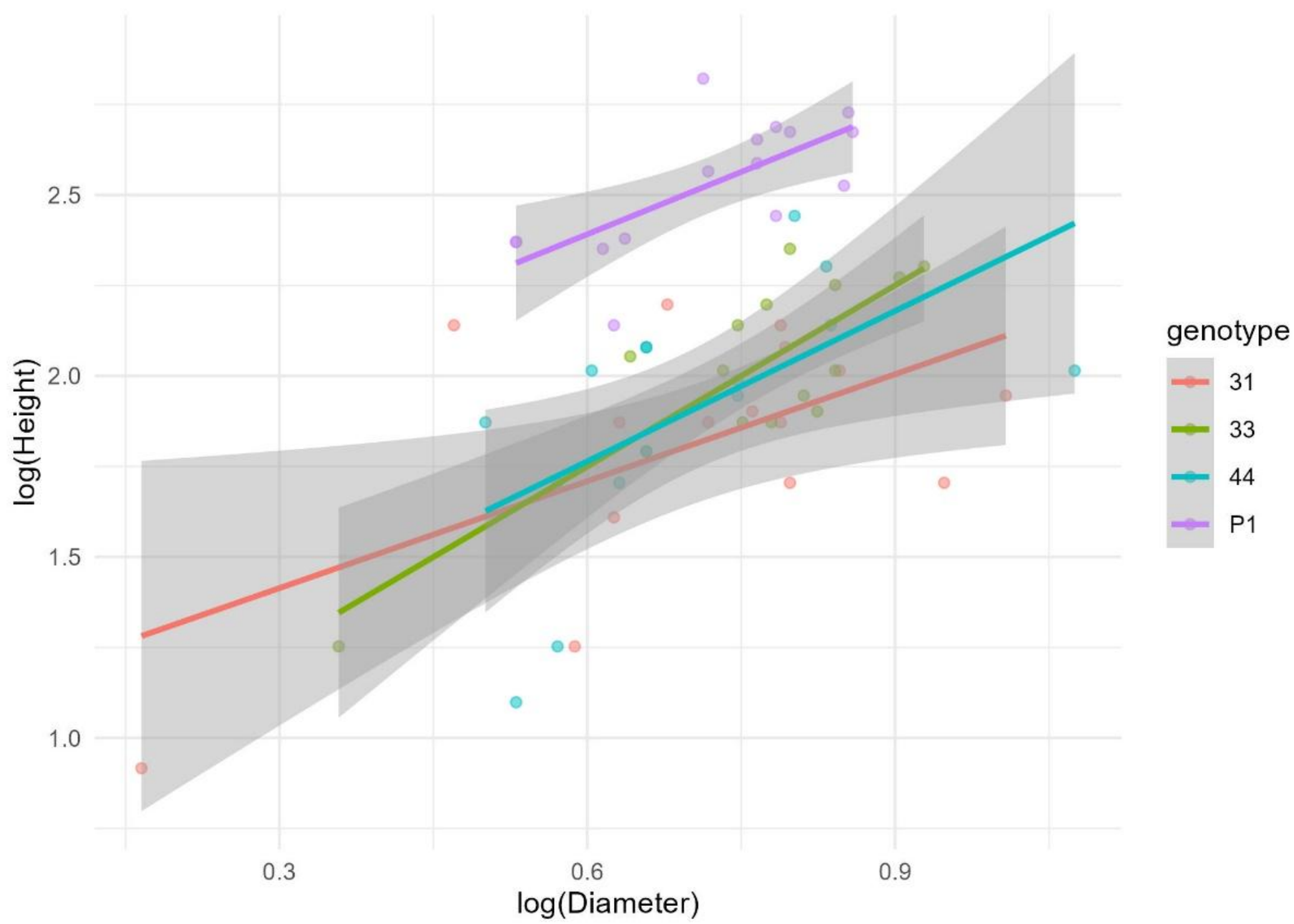

Supplement: Supplementary file 1 [file DataSheet1.pdf]
